# Cyclodextrins for the Delivery of Bioactive Compounds from Natural Sources: Medicinal, Food and Cosmetics Applications

**DOI:** 10.3390/ph16091274

**Published:** 2023-09-08

**Authors:** Stamatia Christaki, Eleni Spanidi, Eleni Panagiotidou, Sophia Athanasopoulou, Anastasia Kyriakoudi, Ioannis Mourtzinos, Konstantinos Gardikis

**Affiliations:** 1 Laboratory of Food Chemistry and Biochemistry, School of Agriculture, Aristotle University of Thessaloniki (AUTH), 54124 Thessaloniki, Greece; stamchri@agro.auth.gr (S.C.); ankyria@agro.auth.gr (A.K.); mourtzinos@agro.auth.gr (I.M.); 2 APIVITA SA, Industrial Park, Markopoulo, 19003 Athens, Greece; spanidi-e@apivita.com (E.S.); panagiotidou-e@apivita.com (E.P.); athanasopoulou-s@apivita.com (S.A.)

**Keywords:** encapsulation, carriers, stability, polyphenols, packaging, supplements, cosmeceuticals

## Abstract

Cyclodextrins have gained significant and established attention as versatile carriers for the delivery of bioactive compounds derived from natural sources in various applications, including medicine, food and cosmetics. Their toroidal structure and hydrophobic cavity render them ideal candidates for encapsulating and solubilizing hydrophobic and poorly soluble compounds. Most medicinal, food and cosmetic ingredients share the challenges of hydrophobicity and degradation that can be effectively addressed by various cyclodextrin types. Though not new or novel—their first applications appeared in the market in the 1970s—their versatility has inspired numerous developments, either on the academic or industrial level. This review article provides an overview of the ever-growing applications of cyclodextrins in the delivery of bioactive compounds from natural sources and their potential application benefits.

## 1. Introduction

Natural compounds have been used since ancient times for their beneficial properties and have played a crucial role in drug discovery. Such natural, biologically active compounds are derived from plants, animals or mineral sources and include, among others, vitamins, phenolic compounds, alkaloids and terpenoids. These groups of bioactive compounds possess antitumor, neuroprotective, antioxidant, anti-inflammatory and antimicrobial properties, so they have numerous applications in the medical, pharmaceutical, food and cosmetic industries [1,2,3]. However, despite their biological activities, they are characterized by poor solubility, bioavailability and stability in their bulk form, so their incorporation in various commercial health-related products is challenging. These drawbacks can be resolved through their encapsulation in delivery systems, one of which are cyclodextrins (CDs) [4].

CDs are a group of cyclic oligosaccharides produced from starch, consisting of a hydrophobic interior cavity and hydrophilic exterior. They were first described by Antoine Villiers more than one hundred years ago. In his work, Villiers reported that potato starch fermentation, under certain incubation conditions with *Bacillus amylobacter*, could lead to the recovery of dextrins [5,6]. However, the current term ‘cyclodextrin’ was adopted many years later and is attributed to Friedrich Cramer at the end of the 1940s [7]. Since then, an increasing number of scientific publications has been focusing on studying their structure, physicochemical properties and applications, as well as the development of new CD-based systems (e.g., nanoparticles) [8,9,10].

The most common types of CDs used in the delivery of bioactive molecules are alpha-cyclodextrin (α-CD), beta-cyclodextrin (β-CD) and gamma-cyclodextrin (γ-CD). α-CD is the smallest CD with six glucose units in the ring. It has a relatively small hydrophobic cavity and is commonly used for complexing small lipophilic molecules. β-CD is one of the most widely used CDs in bioactive compound delivery. It consists of seven glucose units in the ring, providing a larger hydrophobic cavity than α-CD. It can form inclusion complexes with a wider range of molecules including both lipophilic and hydrophilic compounds. γ-CD has eight glucose units in the ring, offering an even larger hydrophobic cavity compared to β-CD that can encapsulate larger molecules. Apart from the above three commonly used CDs, their derivatives have been introduced and applied to enhance the drug-binding capabilities of native CDs. In the first case, CDs can be chemically modified by adding methyl groups to the hydroxyl groups on the glucose units through a process called O-methylation. Examples of such CDs include heptakis (2,6-di-O-methyl)-β-cyclodextrin (DIMEB) and heptakis (2,3,6-tri-O-methyl)-β-cyclodextrin (TRIMEB). It is interesting to note that methylation affects the hydrogen bond network of CDs, thus influencing the intermolecular interactions’ strength and the host: guest flexibility. This could offer increased inclusion complex (IC) stability through stronger hydrogen bonds and/or Van der Waals interactions [11]. With another process, called acetylation, hydroxypropyl groups are added to the hydroxyl groups of CDs. So, for example, (2-hydroxy)propylated β-CD (HPβCD) can be produced from β-CD. Similarly to methylation, the degree of substitution (DS) in this case affects the CD’s properties. For example, DS-differentiated HPβCDs present various molecular recognition abilities, while their catalytic abilities also differ regarding organic reactions in water [12]. The selection of a specific CD type for the delivery of bioactive molecules depends on the physicochemical properties of the latter, the desired release profile and the intended route of administration [10].

**Figure 1:**
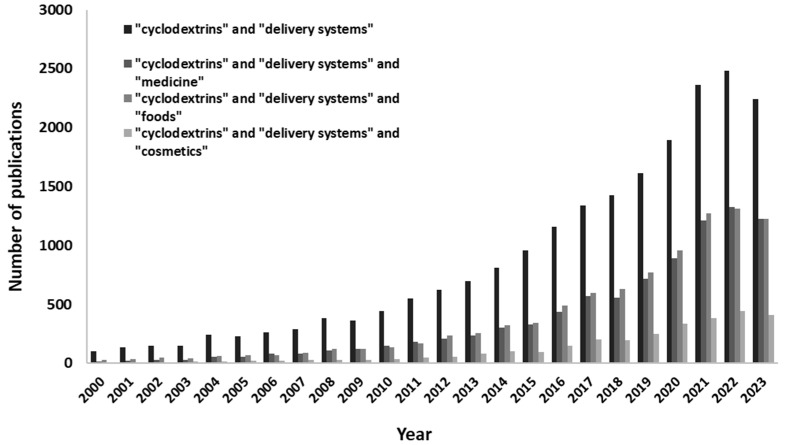
Trends in the number of publications that contain the words “cyclodextrins” and “delivery systems”; “cyclodextrins” and “delivery systems” and “medicine”; “cyclodextrins” and “delivery systems” and “food”; and “cyclodextrins” and “delivery systems” and “cosmetics” in the title, abstract or keyword fields, in the period 2000–2023 (Science Direct search carried out on 28 August 2023).

Introducing the word “cyclodextrins” and “delivery systems,” it is evident that the interest of the scientific community toward CD research is continuously increasing (Figure 1) (Science Direct, last accessed on 28 August 2023). As shown in Figure 1, the majority of the scientific articles are related to medicinal and food applications, whereas publications that contain a combination of the keywords “cyclodextrins” and “delivery systems” and “cosmetics” are more limited. In the present review article, the incorporation of natural bioactive compounds in CDs or their derivatives along with their applications in medicine, food and cosmetics are presented and critically discussed.

## 2. Medicinal Applications

### 2.1. Legislation

CDs are exploited in medicine either as medicinal compounds or as excipients in drug formulation that confer enhanced solubility and stability properties [13]. The first medical product that took advantage of the stability effects of CDs was launched in the Japanese market in 1976 (Prostar-mon E™ sublingual tablet) [14] and, recently, at least 130 CD-formulated drugs have been listed [15]. In Japan, α-CD, β-CD and γ-CD are considered natural products and they are used in the pharmaceutical industry with few restrictions. The natural α-CD, β-CD and γ-CD are included in the GRAS (Generally Recognized as Safe) list of the Food and Drug Administration (FDA). In particular, α-CD and γ-CD have no restrictions on their consumption while there are limits on the oral intake of β-CD. In Europe, the pharmaceutical applications of CDs are regulated by the European Medicines Agency (EMA). The EMA has published a report regarding the appropriate uses of CDs in pharmaceutical products [16].

### 2.2. Applications

In this section, the applications of CD ICs with bioactive compounds from natural sources (e.g., polyphenols, alkaloids, terpenoids, flavonoids and essential oils) in the medical field and research are presented (Table 1). The following studies mainly focus on the improvement of in vitro activities of various bioactive compounds with potent pharmacological properties, such as anticancer, anti-inflammatory and neuroprotective activities, after their complexation with CDs.

Several compounds of natural origin have been proposed to exert antitumor activities. One example is ursolic acid, a triterpenoid found in human diet and medicinal herbs. Complexation of ursolic acid with HPβCD showed an enhanced in vitro antiproliferative activity on melanoma cell lines compared to the pure compound [17]. ICs were also efficient regarding the improvement of antitumor activities of other two terpenoids, namely, saikosaponin-d and betulinic acid. Specifically, HPβCD increased the solubility of saikosaponin-d and improved its antitumor activity in a cell line of human skin squamous cell carcinoma [18]. Regarding betulinic acid, its microencapsulation in β-CD exerted anti-proliferation effects on human breast cancer cells [19]. Similarly, HPβCD enforced the antitumor activity of the marine-derived carotenoid fucoxanthin in human colorectal carcinoma cells in vitro [20]. Camptothecin, and its natural analogue luotonin A, are alkaloids isolated from *Camptotheca acuminata* and are both studied for their anticancer activities. Complexation of these two alkaloids with β-CD and HPβCD enhanced their stability. Moreover, the latter ICs increased the antitumor activity of camptothecin and luotonin A compared to their free forms against a variety of cancer cell lines [21]. In another study, dihydroquercetin (taxifolin), a well-known flavonoid, was complexed with β-CD, resulting in enhanced in vitro antioxidant and antitumor activities against a hepatocellular carcinoma [22]. Similarly, β-CD also improved the cytotoxic activity of the naphthoquinone mansonone G on lung cancer cells [23]. The enhancement of biological activities of these compounds could be attributed to their complexation with CDs. Through IC formation, the bioactive compounds are protected from degradation, so their activities are prolonged. Also, their increased solubility enables their easier penetration in the human cells.

In some cases, more complicated CD-based systems are developed for the encapsulation of natural bioactive compounds. In a study, oxyresveratrol, a stilbenoid found in white mulberry, was encapsulated in a β-CD-based nanosponge. This polymer consisted of 1,1’-carbonyldiimidazole (CDI) and β-CD (β-CD: CDI 1:4) and showed a protective effect regarding oxyresveratrol’s stability during in vitro digestion, as well as stronger cell viability inhibition against prostate and colon cancer cell lines. The researchers reported that the choice of such an encapsulation system provides the ability of even slower release rates compared to simple CD complexation, so the compounds can be used in pharmaceutical products [24]. A similar system has also been used for the encapsulation of ferulic acid, leading to enhanced antiproliferation activities against breast cancer cell lines [25]. Finally, in the field of cancer research, curcumin, a widely known curcuminoid, has been encapsulated in several CD-based systems aiming to enhance its solubility and consequently its antitumor activities. Encapsulation of curcumin in HPγCD liposomes strengthened its cytotoxic activities against osteosarcoma and breast cancer cell lines [26]. Another type of curcumin combination with HPβCD and chitosan microspheres was found to improve the therapeutic index in human colorectal adenocarcinoma cell line [27]. It should be noted that the research conducted in terms of anticancer and antitumor activities of encapsulated natural bioactive compounds in CDs and their systems is quite challenging. Most of the studies report their results in vitro, so it is quite unclear whether these biological activities could be equally effective in vivo. Nevertheless, the current reported results are promising regarding the effectiveness of CD-based systems to enhance the antitumor activities of tested bioactive compounds and further research should be performed in this field.

Beyond the reinforcement of antitumor activities, complexation of bioactive compounds with CDs has been studied for various other potent therapeutic properties, such as anti-inflammatory, anti-allergic, anti-viral and others. Thymoquinone is a natural compound found in the plant *Nigella sativa*. Thymoquinone complexation with HPβCD in non-toxic levels ameliorated its solubility and anti-allergic effects compared to free thymoquinone or the anti-allergic drug, cetirizine [28]. Green propolis supercritical extract (GPSE) encapsulated in γ-CD exhibited anti-inflammatory activity through diet in mouse liver in vivo [29]. Known for its hepatoprotective capacity, the flavonolignan silybin (also known as silibinin), has been studied as a potent therapeutic agent for the treatment of non-alcoholic fatty liver disease (NAFLD). Silybin–HPβCD complex improved gut health though restoration of gut microbiota in high-fat diet (HFD)-fed hamsters [30]. In another study, an improved gastroprotective activity of (-)-linalool was reported in rodents upon complexation of this terpene with β-CD [31]. A recent study revealed an enhanced antiviral effect of epigallocatechin gallate, a catechin abundant in tea, against different viruses, e.g., strains of influenza virus and the coronavirus 229E. The compound formed an inclusion complex with a CD mixture composed of 0.1% β-CD, 0.4% γ-CD, and 0.2% ascorbic acid [32]. The neuroprotective effects of isothiocyanate moringin in an Alzheimer’s disease (AD) in vitro model was reported in another study. Moringin, the major bioactive compound in moringa seeds, was complexed with α-CD. Pretreatment of human neuroblastoma cell line SH-SY5Y (differentiated with retinoic acid) with the complex was able to reduce the production of proteins involved in the AD cell degeneration processes [33].

Apart from the academic research conducted in the field, several patents reflect the significance of CD-based ICs with natural bioactive compounds. In fact, the patented formulations are based on already known bioactive compounds, since they were mentioned in the paragraphs above. Indicatively, one patent refers to the improvement of curcumin’s stability through complexation with HPβCD [34], while one other describes the preparation of a bio-compatible CD complex with triterpenes, such as ursolic acid [35]. A very interesting patent deals with the complexation of betulinic acid with CD for its subsequent application in the treatment of Alzheimer’s disease [36]. Moreover, ICs of lycopene, quercetin and curcumin with HPβCD have been patented as pharmaceutical formulations for the preparation of products treating prostatitis [37]. It is clear that the interest of researchers and industry mainly focuses on bioactive compounds that possess anti-inflammatory activities so they can be used in pharmaceutical products. In addition, the compounds in the patented formulations are some of the most widely used in the literature, thus underlining their importance and justifying the interest of the scientific community towards their investigation.

**Table 1:** In vitro and in vivo studies of CDs complexed with natural bioactive compounds with potent medicinal applications.

CD Type	Bioactive Compound	Improved Characteristics	Biological Study	Test Subject of In Vitro/In Vivo Study	Reference
HPβCD	Ursolic acid	Stability	Antitumor activities	Melanoma cell lines (A375, B16 4A5 and SK-Mel 2)	[17]
HPβCD	Saikosaponin-d	Solubility	Antitumor activities	Squamous carcinoma cell line (HSC-1)	[18]
β-CD	Betulinic acid	-	Antitumor activities	Breast cancer cell line (MCF7)	[19]
HPβCD	Fucoxanthin	Solubility and stability	Antitumor activities	Colorectal carcinoma (CRC) cells (HCT116 and Caco-2)	[20]
β-CD and HPβCD	Camptothecin	Stability and solubility	Antitumor activities	Breast cancer (AREc32), lung cancer (H-23), hepatic carcinoma (HepG2), ovarian carcinoma (A2780) and neuroblastoma (SH-SY5Y) cell lines	[21]
	Luotonin A	Stability and solubility			
β-CD	Dihydroquercetin	Solubility	Antioxidant and antitumor activities	Hepatocarcinoma cell line (HepG2)	[22]
β-CD	Mansonone G	Solubility	Antitumor activities	Lung cancer cells (A549)	[23]
β-CD: CDI 1:4	Oxyresveratrol	Dissolution	Antitumor activities	Prostate (PC-3), colon (HT-29 and HCT-116) cell lines	[24]
CD-NSs	Ferulic acid (FA)	Stability	Antitumor activities	Breast cancer cell lines (MCF7 and 4T1)	[25]
γ-CD liposomal nanoparticles	Curcumin	Solubility	Antitumor activities	Osteosarcoma (KHOS) and breast cancer (MCF-7) cell lines	[26]
HPβCD	Thymoquinone	Solubility	Antiallergic effects	Rat basophilic leukemia cell line (RBL-2H3)	[28]
γ-CD	Green propolis supercritical extract (GPSE)	-	Anti-inflammatory activities	Female C57BL/6NRj wild-type mice (liver)	[29]
HPβCD	Silybin (silibinin)	Solubility	Restored the gut microbiota and intestinal integrity	Hamsters	[30]
β-CD	(−)-linalool	Solubility and stability	Gastroprotective effect	Mice	[31]
β-CD, γ-CD	Epigallocatechin gallate	Stability	Antiviral effect	Influenza virus and HCoV-229E	[32]
α-CD	Moringin (MOR)	-	Neuroprotection	Neuroblastoma cells (SH-SY5Y) exposed to amyloid beta peptide	[33]

## 3. Food Applications

The applications of CDs as carriers for bioactive compounds and their incorporation in various food products has been widely studied in the literature. Although CDs were initially tested and applied as drug delivery systems [8], their characteristics enabled food researchers to also widen their applications in various food matrices. Either as carriers for single bioactive compounds or as carriers for crude essential oils (EOs), CDs are effectively applied in many food products. Usually, such products are fresh, easily perishable ones (e.g., fruits, juice, fresh meat) since their composition (e.g., high moisture content) enables their microbiological and physicochemical degradation [38]. As delivery systems, CDs offer a variety of advantages in the final food product, mainly by preserving or enhancing the activities of the entrapped bioactive compounds. Firstly, the formation of inclusion complexes with CDs promotes the easier solubility of bioactive compounds in aqueous matrices [39]. This is of key importance since most of the food products that require preservation are water-based systems. In addition, encapsulation in CDs offers increased stability to the bioactive compounds as they are protected from adverse conditions (e.g., oxygen, heat) during food processing and storage. In this way, their biological activities are prolonged [40,41]. Especially for volatile compounds such as EOs and their constituents, which are most commonly applied as natural antimicrobial and antioxidant agents in foods, encapsulation in CDs decreases their evaporation rate and promotes their controlled and sustained release in the food matrix [42]. Moreover, one of the main advantages of bioactive compound encapsulation in CDs is the masking of their intense odor and taste, thus decreasing their negative effect on the organoleptic properties of foods [43].

The most widely studied and applied CD in food products is β-CD due to its affordability and compatibility with a variety of molecules [44]. β-CD is essentially odorless and white/whitish, while its aqueous solution is clear and colorless. Although β-CD is moderately soluble in water, it can easily be dissolved in warm water, whereas its solubility in ethanol is low (Regulation (EU) No 231/2012) [45]. As an approved food additive, β-CD is listed in Annexes II and III of Regulation (EC) No. 1333/2008, under the code E 459 [46]. Derivatives of β-CD, such as HPβCD, are also used. As reported by [47], CDs do not passively diffuse through biological membranes due to their hydrophilic nature, characterized by numerous hydrogen bond donors and acceptors and very low octanol–water partition coefficients. In addition, CDs present very low bioavailability when administered orally; β-CD, α-CD and their derivatives are mainly digested by bacteria in the colon, and γ-CD is completely digested in the gastrointestinal tract. In this way, they are practically nontoxic, and no CD accumulation is observed in healthy individuals with normal kidney function, even at high doses, whereas caution is recommended in the case of severely renally impaired patients [47].

Many studies in the literature have evaluated the effect of IC incorporation, between CDs and bioactive compounds, in the final food product in terms of microbiological, oxidative, color stability, and organoleptic characteristics. Guest moieties commonly studied are EOs (e.g., clove, rosemary, lemongrass) and their constituents (e.g., cuminaldehyde, thymol, citral) and phenolic compounds that are mainly hydrophobic (e.g., resveratrol, oxyresveratrol, ferulic acid, gingerols, curcumin). All the above are well-known natural compounds with important antioxidant and antimicrobial activities. Apart from direct incorporation of ICs in the food products, there are also indirect applications via active food packaging [48]. This method of preservation has been widely studied through the last years, where edible materials can be used to form a film/coating incorporating various bioactive compounds and applied externally in the food product of interest. In this way, the respective products are protected from external factors and can be preserved for a longer period of time (extension of shelf-life) [49]. Applications of active food packaging containing CD-based ICs or ICs directly incorporated in different food systems and their main effects are presented in Table 2 and Table 3, respectively.

**Table 2:** Applications of active packaging containing CD-based ICs in different food systems and their main effects.

CD Type	Bioactive Compound/Guest Moiety	IC ^1^ Preparation Methods	Packaging Material	Food System/Model	Effects in the Final Product	Reference
β-CD	Cinnamaldehyde (CIN)	Mixing and freeze-drying	Non-woven polyethylene terephthalate (PET)	Cold fresh pork	Packaged pork samples with the highest tested CIN concentration were preserved for 11 days under refrigerated storage compared to control samples (7 days).	[50]
Methyl-β-CD	*Satureja montana* L. essential oil (SEO)	Mixing, ultrasonication and freeze-drying	Soy soluble polysaccharide (SSPS) hydrogel	Meat slices	Methyl-β-CD/SEO-SSPS hydrogel effectively reduced *S. aureus* counts by 3.5 log CFU/g after 7 days of storage at 4 °C.	[51]
β-CD	Octyl gallate (OG)	Co-precipitation and freeze-drying	Chitosan film	Fresh fruits vegetables (blueberries and asparagus)	Lower weight loss was reported in coated asparagus samples containing 0.5%, 1.0% and 2.0% β-CD/OG (3.87%, 3.12% and 2.85%, respectively), compared to control (7%) after 25 days storage at 4 °C. TVC ^2^ was maintained close to the initial 10^2^–10^3^ CFU/g in the coated asparagus samples compared to control (10^7^ CFU/g) after 25 days of storage at 4 °C.	[52]
					Coated blueberries with films containing 1.0% and 2.0% β-CD/OG presented lower weight loss (2%) compared to control (7%) after 25-day storage at 4 °C. Films containing 2.0% β-CD/OG effectively preserved freshness in blueberries with a 6% rotting rate compared to control (20%).	
β-CD	*Trans*-cinnamaldehyde (TC) and citral (CI)	Co-precipitation and vacuum-drying	Ethylene vinyl alcohol copolymer (EVOH) film	Beef	Shelf-life of EVOH-β-CD-CI and EVOH-β-CD-TC coated samples was extended about 4 days at 4 °C, compared to control and coated samples without ICs.	[53]
β-CD	Curcumin (Cur)	Mixing and freeze-drying	κ-Carrageenan (κ-Car) film	Chilled pork	Extension of chilled pork shelf life from 4–5 days to 10 days with application of κ-Car-β-CD-Cur film combined with light treatment, compared to pure κ-Car film and other treatments.	[54]
β-CD	Lemongrass essential oil (LEO)	Co-precipitation and drying	Chitosan–gelatin (CS-Gel) coating	Fresh cherry tomatoes	CS/Gel coating with 7% β-CD/LEO presented high antibacterial activity against *P. aurantiogriseum* in cherry tomatoes artificially during 20 days of cold storage at 8 °C.	[55]
α-CD	Benzyl isothiocyanate (BITC)	Mixing, ultrasonication and vacuum freeze-drying	Chitosan (CS) film	Beef	CS-α-CD-BITC-coated beef samples presented lower TVC, TVB-N ^3^ and TBARS ^4^ values and higher overall acceptability score, compared to PET- ^5^ and CS-coated samples after 12 days of refrigerated storage.	[56]

^1^ ICs: Inclusion complexes, ^2^ TVC: total viable count, ^3^ TVB-N: total volatile base nitrogen, ^4^ TBARS: thiobarbituric acid reactive substances, ^5^ PET: polyethylene terephthalate.

Films/coatings incorporating ICs have been applied in meat products (beef, pork) for preservation (Table 2). Zhou et al. reported the extension of refrigerated storage of fresh pork packaged with non-woven PET containing CIN-β-CD ICs by 4 more days compared to the control (non-packaged samples) [50]. Similar results were reported by Wu et al., where κ-carrageenan films containing Cur-β-CD ICs, combined with light treatment, extended the shelf-life of chilled pork by 5 days, compared to pure κ-carrageenan films (no extension) [54]. Chitosan-based films with ICs of benzyl isothiocyanate, a compound found in the plants of the *Brassicaceae* family, and α-CD effectively preserved beef samples after 12 days of refrigerated storage. These coated samples were less oxidized after storage (lower TVB-N and TBARS values) and more microbiologically stable (lower TVC) compared to samples coated with pure chitosan films or PET [56]. Chen et al. used films from ethylene vinyl alcohol copolymer (EVOH) incorporating ICs of β-CD with two essential oil constituents, namely, *trans*-cinnamaldehyde and citral, for the preservation of beef [53]. The results showed that EVOH-coated beef samples containing ICs were preserved for 4 more days in refrigerated storage compared to both non-coated samples and EVOH-coated samples without ICs. It can be concluded that encapsulation of bioactive compounds in the cavity of CDs prior to their incorporation in the film/coating-forming solution enhances their solubility and preserves their biological activities. However, for comparison reasons, it would be also useful to prepare films/coatings containing the pure compounds (without CDs). In this way, an overall conclusion can be drawn regarding not only the extension of biological activities of bioactive compounds, but also the other advantages that CDs offer (sustained release, odor masking, etc.).

**Table 3:** Applications of CD-based ICs in different food systems and their main effects.

CD Type	Bioactive Compound/Guest Moiety	IC ^1^ Preparation Methods	Food System/Model	Effects in the Final Product	Reference
β-CD	Cuminaldehyde (CUM)	Ultrasonication, cold nitrogen plasma (CNP) treatment and freeze-drying	Vegetable juices (tomato and cucumber)	CNP-treated ICs decreased the *E. coli* O157:H7 population from 3.5 log CFU/mL to 2.51 (12 °C) and 1.29 log CFU/mL (4 °C) on cucumber juice, and to 2.58 (12 °C) and 1.33 log CFU/mL (4 °C) on tomato juice after 3 days of storage, compared to control (no added ICs).	[57]
β-CD	Ferulic acid (FA)	Crosslinking of β-CD with diphenyl carbonate (nanosponges preparation), agitation and freeze-drying	Pomegranate juice	Highest TPC ^2^ and antioxidant activity of pomegranate juice treated with FA-CD-NSs ^3^ containing 500 mg/L FA was reported after 30 days of storage at 4 °C compared to control and samples containing free FA. Total anthocyanins were better stabilized in pomegranate juice treated with FA-CD-NSs containing 250 mg/L FA after 30 days of storage at 4 °C, compared to control and samples containing free FA, through co-pigmentation effect.	[58]
β-CD	Clove essential oil (CEO)	β-CD-metal organic frameworks (β-CD-MOFs) preparation through methanol vapor diffusion, mixing and freeze-drying	Chinese bacon (preserved meat product)	The lowest MDA ^4^ and POV ^5^ values were reported in Chinese bacon preserved with CEO-β-CD-MOFs in all tested concentrations, after 3 days of preservation and 15 days of fermentation compared to control, samples containing free CEO or BHT ^6^.	[59]
β-CD	Fish oil (FO)	Homogenization for emulsion formation and ultrasonication	Yogurt	FO-IC-treated yogurt presented greater syneresis reduction and lower POV values, but higher DHA ^7^ and EPA ^8^ content, after 21 days of storage at 4 °C compared to control and samples containing free FO.	[60]
				FO-IC-treated yogurt was significantly better accepted regarding sensory characteristics compared to the free-FO-treated one.	
γ-CD	Resveratrol (RSV)	Mixing, snap-freezing and freeze-drying	Lemon juice	RSV encapsulation in γ-CD improved its solubility in lemon juice by nine times compared to free RSV (43.1% and 4.8% dissolution, respectively) at day 0. Higher RSV content was reported in γ-CD-RSV-treated lemon juice after 28 days of storage under dark conditions (room temperature or 4 °C).	[61]
HPβCD	Apple polyphenols (AP)	Mixing and freeze-drying	Lamb	Frozen-stored lamb treated with 1.6 mg/mL AP/HPβCD-ICs presented the lowest carbonyl content (protein oxidation parameter) and improved muscle tissue structure after 40 days of storage compared to control and other tested IC concentrations.	[62]
γ-CD	Epigallocatechin-3-gallate (EGCG)	Co-precipitation and freeze-drying	Shrimp surimi products	γ-CD-EGC-treated shrimp surimi products were better preserved regarding lipid oxidation phenomena and browning effects from EGCG oxidation after 5 weeks under refrigerated storage compared to control and free-EGCG-treated samples.	[63]
γ-CD	Gingerols (GINs)	Co-precipitation and drying	Yogurt	Lower ΔΕ in γ-CD-GIN-treated yogurt compared to the free-GIN-treated one regarding L*, a* and b* color parameters (control used as reference).	[64]
				γ-CD-GIN-treated yogurt presented higher ABTS radical scavenging activity compared to control and the free-GIN-treated one.	
HPβCD	Thymol (Th)	Ultrasonication and freeze-drying	Tomatoes	A 66.55% lower disease incidence from *B. cinerea* in tomato samples treated with 30 mg/mL HPβCD-Th-ICs compared to control after storage for 3 days at 25 °C.	[65]
β-CD and HPβCD	Oxyresveratrol (Ox)	Agitation and spray-drying	Grape juice	Ox-β-CD- and Ox-HPβCD-treated samples, combined with ascorbic acid, presented the lowest L* and ΔΕ value differences (compared to 0 h) after 24 h of storage at room temperature, indicating an anti-browning effect.	[66]
β-CD	Rosemary essential oil (REO)	Co-precipitation and drying	Tomato juice	In REO-β-CD-treated tomato juice, the population of *S. pastorianus* decreased from 5.5 log CFU/100 mL (day 0) to 2 log CFU/100 mL after 15-day storage at 5 °C, and this difference was significantly higher compared to control and free-REO-treated samples.	[67]

^1^ ICs: inclusion complexes, ^2^ TPC: total phenolic content, ^3^ NSs: nanosponges, ^4^ MDA: malondialdehyde, ^5^ POV: peroxide value, ^6^ BHT: butylated hydroxytoluene, ^7^ DHA: docosahexaenoic acid, ^8^ EPA: eicosapentaenoic acid.

In contrast to the active food packaging containing ICs, which has been mainly applied to meat products, pure ICs containing bioactive compounds have been commonly used for juice preservation (Table 3). This is expected, since ICs are in solid, water-soluble form (powder), thus enabling their solubilization in aqueous matrices. In addition, due to the high amount of sugars and water present in juices, the incorporation of antioxidant and antimicrobial agents is crucial for their preservation during storage. CUM-β-CD ICs that were treated with cold nitrogen plasma were added in vegetable juices for evaluation of their antibacterial activity against *E. coli* O157: H7. The results showed that ICs were effective against *E. coli*, since its population decreased both in 4 °C and 12 °C compared to control samples (no added ICs). The decrease was bigger in the case of 4 °C, implying a synergy between cold storage and the antibacterial agent, cuminaldehyde [57]. Garcia-Sotelo et al. evaluated the application of REO-β-CD ICs in tomato juice regarding the antimicrobial activity against the yeast *S. pastorianus* [67]. The population of *S. pastorianus* decreased by 3.5 log CFU/100 mL in the IC-treated samples after 15 days of storage at 5 °C, whereas control and samples treated with free (non-encapsulated) REO did not present the same population decrease. In a study by Amani et al. [58], the incorporation of FA-β-CD ICs in pomegranate juice was studied in terms of anthocyanin content, among other factors. The results showed that, in the presence of ICs containing 250 mg/L FA, total anthocyanins were better stabilized in the juice after 30 days of storage at 4 °C compared to control and samples containing free FA due to a co-pigmentation effect. Oxyresveratrol, a natural stilbenoid with high antioxidant activity, was complexed with β- and HPβCD and added in grape juice for elimination of browning phenomena responsible for the product’s degradation [66]. The application of both ICs and ascorbic acid in grape juice effectively preserved the color (in terms of lightness L* and ΔΕ) after 24 h of storage at room temperature.

The importance of CDs as carriers for bioactive compounds is also highlighted by the number of patented works regarding applications of such complexes in various food systems. Indicatively, the preparation of ICs of at least one CD, or derivatives, complexed with terpene glycosides (e.g., steviol glycosides) for the production of beverages in the food industry has been patented [68]. The complexation of such glycosides with CDs is expected to increase their solubility in the beverage matrix. Another patented invention regarding non-alcoholic beverages focuses on the reduction and future replacement of conventional preserving agents in such products by suggesting an antimicrobial system based on CD complexes with natural compounds (e.g., limonene, cinnamaldehyde) that will not increase the beverage pH higher than 7.5 [69]. In another patent, the development of ICs of sucralose (sweetener) with CD is described [70]. Later on, another patented invention focused on the development of gum containing the above ICs, aiming to preserve the activity of sucralose, i.e., the sweet taste of gum, throughout chewing [71]. One more very interesting, patented invention describes the production of CD complexes with colorant-eliminating agents and their incorporation in confections (e.g., gum) in order to remove colorants from teeth surfaces [72]. The researchers suggest the incorporation of such ICs in the final stages of gum production. The preparation and preservation of a bakery product containing ICs of natamycin with CD is presented in another patented invention. For antimicrobial effectiveness, the ICs should be applied on the surface of the bakery product and the amount of natamycin should range from 0.1 pg/cm^2^ to 7.0 pg/cm^2^ [73]. Last but not least, the development of ICs between carotenoids, such as astaxanthin or lycopene, with CDs for the improvement of their storage stability compared to carotenoids in their free form is described in another patent [74]. The researchers highlight the ability of the ICs containing carotenoids to also improve pigmentation in animal tissues (e.g., salmon), and be used as feed or food supplement. It can be observed that the CD-based complexes can be used in various food products. The interest of researchers is basically focused on the delivery of bioactive compounds that can act as preservatives, while in most cases the ability of CDs to eliminate the taste, aroma or color of bioactive compounds is taken into consideration for the development of novel formulations.

## 4. Dietary Supplements Applications

The advantages of CDs regarding improved solubility, absorption and bioavailability of the entrapped bioactive compounds have favored their applications in dietary supplements. Similarly to food applications, CDs have been employed as carriers for sensitive, easily-degradable, lipophilic compounds that exert important biological activities and should be preserved throughout the gastrointestinal tract (GT) until their absorption in the human body [75]. An overview of literature studies regarding applications of CD-based inclusion complexes in dietary supplements is presented in Table 4. In all cases, the bioavailability of bioactive compounds of interest was increased, highlighting the ability of ICs to effectively protect and deliver them through the GT. For this reason, commercial dietary supplements containing CD complexes have already been developed.

A dietary supplement with R-alpha lipoic acid complexed with CDs (R-ALA Cyclodextrin Complex Tablets^®^) is used for mitochondrial function enhancement and promotion of metabolic function and antioxidant activity in a suggested dose of 500 mg/day. The complexation improves both the stability and bioavailability of R-ALA. Turkesterone, a steroid hormone of *Ajuga Turkestanica*, and ecdysterone, a steroid hormone from *Cyanotis arachnoidea* root extract (similar molecular structure to testosterone), are used as dietary supplements for improvement of athletic performance, promotion of lean muscle growth, support of the immune system and muscle repair and recovery. Complexation of these compounds with HPβCD offers maximum absorption and increased potency (Ecdysterone Complexed With Hydroxypropyl-β-Cyclodextrin^®^, Turkesterone Complexed With Hydroxypropyl-β-Cyclodextrin^®^). Dietary supplements of curcumin complexed with γ-CD (Curcumin Extrakt 45^®^) and manuka honey complexed with α-CD (MGO^TM^ 400 Manuka honey^®^) are mainly used for their anti-inflammatory and antioxidant activities. In both cases, the absorption and bioavailability of the encapsulated bioactive compounds have increased. γ-CD was also used for the encapsulation of ubiquinone (CoQ10), an important coenzyme of the human body, in a dietary supplement that promotes the replenishment of CoQ10 levels in the body and provides energy (Co-Enzyme Q10+^®^). It was reported that the CoQ10 absorption was improved by 18× through complexation with γ-CD compared to free CoQ10.

Apart from commercial products, there are also patented inventions based on applications of ICs in dietary supplements. Regarding CoQ10 again, a patent describes the preparation of water-soluble ICs of the compound with β-CD that aims to increase the water solubility, bioavailability and biological activities of CoQ10 [76]. The development of a dietary supplement containing nano-ICs of carotenoids with γ-CD is pending patenting [77]. The researchers propose an easy mixing method for the dietary supplement preparation, which also includes fish oil and beeswax, apart from the ICs. In the same line, the water solubility of trans-resveratrol, an antioxidant polyphenol, is improved through complexation with β-CD [78]. The ICs are proposed as useful phytotherapeutic ingredients for incorporation in nutraceutical (pharmaceutical and/or dietary) supplements. The preparation of nutraceutical tablets containing ICs of β-CD with fatty acids (e.g., omega-3, omega-6) for oral administration has also been recently patented [79]. The respective tablets are proposed for the prevention or treatment of cardiovascular-system-, skin- or bone-related disorders.

**Table 4:** Applications of CD-based ICs in dietary supplements and their main effects.

CD Type	Bioactive Compound/Guest Moiety	Targeted Disease/Health Problem	Effects/Key Findings	Reference
γ-CD	Vitamins D3 and E	Cystic fibrosis (with pancreatic insufficiency)	Improved bioavailability of fat-soluble vitamins D3 and E	[80]
HPβCD	Curcumin and piperine (CUR-PIP)	Diseases of infectious or neurodegenerative etiology	Improved membrane permeability, solubility and antimicrobial and antioxidant activity of CUR-PIP was reported after complexation with HPβCD. The system presented enhanced inhibitory activities against butyrylcholinesterase and acetylcholinesterase.	[81]
β-CD	Oregano essential oil (OEO)	Intestinal parasitic infection	Lipid-based nutrient supplement (LNS) containing β-CD-OEO inclusion complexes (27.2 mg/20 g LNS) was stable through the gastrointestinal phases and no differences were reported in sensorial characteristics compared to control.	[82]
β-CD	Diosgenin (DIO)	Hyperlipidemia, hyperglycemia, reduced skin thickness	Highly improved oral bioavailability of β-CD-DIO complexes and skin distribution of diosgenin compared to oral administration of free diosgenin.	[83]

## 5. Cosmetics Applications

In the cosmetics industry, products usually appear in conventional creams, gels, lotions and solid dosage forms. The current needs and requirements of this industry have led to more intense research, development and application of new formulas based onnano-encapsulation and micro-encapsulation techniques. The difficulty of delivering bioactive ingredients to the skin, mainly due to the strong barrier of the *stratum corneum* (SC), requires new delivery systems to create products/formulas with superior technological performance [84,85,86].

In the early 1980s, the first review articles were already mentioning the potential application of CDs in cosmetic and personal care products. Several advantages have already been reported with the use of CDs in cosmetic products, such as the increase in solubility and the rate of solubilization, change in the viscosity or the use of more natural solvents by avoiding the use of organic solvents. In addition, CDs protect the guest bioactive components against hydrolysis or oxidation, degradation induced by heat or light, volatile ingredient loss and reactions with other cosmetic ingredients. The reduction or even elimination of undesired characteristics such as taste or odor and hygroscopicity is one of the advantages of using CDs. The effect of CDs in reducing the microbiological load was also observed since they do not constitute a nutrient medium. Furthermore, encapsulation in CDs offers improvement in handling bioactive compounds, with oily or liquid substances transformed to a solid form, and increases the stability of emulsions and thermal stability of oils [85,87,88,89]. In recent decades, the application of CDs for skin use has been particularly widespread [90] in products aimed at sun protection, wound healing, acne, psoriasis and dermatitis as well as for use in deodorants and shampoos [84].

Bioactive compounds from natural sources are used in the cosmetic industry as they most often present a better safety profile than synthetic ones [91]. Despite having scientifically documented biological activities, they face challenges with their handling and therefore their use and activity is limited. Some of the issues improved with the use of CDs are their bioavailability, solubility and stability [91].

Many bioactive compounds from natural sources have been evaluated for potential use in the cosmetic industry (Table 5). For example, three different types of CDs were used (HPβCD, M-β-CD and HPγCD) to increase the shelf life, antimicrobial properties and loading capacity of linalool for potential applications in personal care and cosmetics [92,93,94]. Encapsulation with β-CD improves the antimicrobial and antioxidant activity of *Lippia graveolens* essential oil for potential use in cosmetics [95]. *Celastrus paniculatus* seed oil was loaded in HPβCD to increase skin penetration and stability [96]. Total phenolics, phenolic acids and flavonoids from *Helichrysum italicum* were loaded in HPβCD, enhancing a metabolite with anti-inflammatory properties as well as anti-elastase and anti-collagenase activities, making it able to use in the cosmetic industry [97]. Also, the use of CDs has been investigated for the separation of bioactive targets for potential use in cosmetics, such as isolating glabridin from a widely used plant in cosmetics, *Glycyrrhiza glabra*, with the help of β-CD and SBE-β-CD [98].

There are several inventions on cyclodextrin entrapment of natural bioactive compounds for cosmetic use. Indicatively, silymarin from the plant *Silybum marianum* L. is entrapped in SBE-β-CDs to prevent skin aging, reduce oxidative stress in skin cells, decrease facial redness in rosacea-prone skin and increase collagen production [107]. In another patented invention, different types of CDs (α-CDs, β-CDs, γ-CDs, HPβCDs, and HPγCDs) are used to improve the solubility and stability of polyphenol mangiferin from the plant *Mangifera indica* L. (mango) [108]. HPβCD was used to increase the solubility of the bioactive components of propolis and increase the efficiency of the extraction, creating a more stable extract rich in antioxidants enriching face and body cosmetic products [109].

A very interesting emerging field is focused on the use of CDs as components of advanced drug delivery systems that are complemented by other particles. In such applications, CDs may be used as encapsulation efficiency enhancers and controlled-release modulators. In the following two examples, CDs were used along with liposomes in order to form a single delivery system with advanced properties. By using HPβCD along with liposomes, a stable colloidal dispersion system with loaded propolis bioactive compounds (polyphenols) was formed. A percentage of 25% up to 60% of the encapsulated propolis compounds are released within 8 h, while total release takes place within a day. The application of this system on primary human skin fibroblasts (NHDF) results in a 100% increase in viability over the control sample, representing an increase in adenosine triphosphate (ATP) therein [110]. In another patent, HPβCD was used to prepare a colloidal stabilization system and controlled release of royal jelly ingredients. CD encapsulates the polyphenols and enhances the system’s penetration into the skin, and its combination with liposomes allows the controlled release of the ingredients [111]. The combination of CD complexes and liposomes for the development of product formulations that are more easily absorbed by the skin, while retaining the biological activity of the encapsulated compounds, perhaps reflects the current market needs. In addition, the competitiveness of the cosmetic industry renders the continuous search and development of novel, natural products crucial. This fact imposes a challenge on researchers to come up with new combinations of delivery systems.

The main concern for the selection of CDs in cosmetics is their safety during their use. Studies have shown contradictory results for skin safety using CDs [112], but the only group of CDs that may affect skin barrier properties are the methylated derivatives [113,114,115], though it depends on their concentration. Generally, natural CDs have been shown to be compatible with the skin and are considered non-irritating and safe for dermal use, as they do not cause disruption of the SC [84,116,117].

## 6. Conclusions

CDs have emerged as remarkably versatile carriers for delivering bioactive compounds derived from natural sources in medicinal, food and cosmetic formulations. Their primary capability lies in their capacity to enhance the solubility, stability and bioavailability of these natural bioactive compounds. This attribute significantly contributes to amplifying therapeutic efficacy in medical applications, driving advancements in functional foods, and fostering innovation in cosmetic formulations. The integration of CDs within these sectors not only promises the development of advanced delivery systems but also unlocks the full range of benefits offered by bioactive compounds sourced from nature. By encapsulating these compounds, CDs facilitate sustained and targeted release, thereby magnifying their impact and expanding their potential uses. Consequently, the convergence of CDs with these industries paves the path for various delivery mechanisms, fully harnessing the multifaceted benefits provided by bioactive compounds derived from natural sources. This integration not only expands the horizons of medical treatments but also holds the potential to revolutionize dietary practices and redefine approaches to cosmetic applications. Although the research around CDs is not a novel field, their ability to be employed in various fields as well as the quite limited number of in vivo studies regarding bioavailability of encapsulated compounds, coupled with the current trend of increasing use of natural bioactive compounds, will ensure the interest around these carriers continues to grow.

## Figures and Tables

**Table 5:** Applications of CD-based ICs in cosmetics and their main effects.

CD Type	Bioactive Compound/Guest Moiety	System/Model	Effects/Key Findings	Reference
β-CDs and HPβCD	Linalool	Gel formulations	Increased the water solubility, improved handling of raw materials (liquid fragrance material to be turned into powder). Controlled release of bioactive compounds and their stability within the formula.	[92]
HPβCD	*Celastrus paniculatus* seed oil (CPSO)	Serum and gel base	Physical stability of formulations containing CPSO with HPβCD after 3 months of storage, with the percentage of oleic acid maintained above 80% of the initial amount. Higher skin penetration of oleic acid is shown compared to other formulations. The formula exhibits appropriate viscosities for use in cosmetic products.	[96]
β-CDs	Tea tree oil	Cosmeceutical facial creams	Stable formulas mainly in rheological properties	[99]
HPβCD	Royal jelly	Cosmeceutical facial creams	Encapsulates the sensitive components of royal jelly (10-HDA), eliminating its stability disadvantages while at the same time allowing time-controlled release which could prove useful for skin applications as indicated by the in-cell experiments	[100]
HPβCD and MβCD	Isoflavones (formononetin and biochanin A)	Hydrogel formulations	Significant increase in the penetration of isoflavones (formononetin and biochanin A) into the epidermis and dermis with the use of HPβCD in hydrogels, with potential use in cosmetic formulations for the prevention of skin aging	[101]
HPβCD	Propolis	Cosmeceutical facial creams	Encapsulation of propolis polyphenols in a physicochemically stable system with a controlled release rate. It maintains the antioxidant, anti-mutagenic and anti-aging properties of propolis polyphenols at levels similar to a methanolic extract	[102]
β-CD	Curcumin	Semisolid Oil in Water (O/W) emulsions	Formulas containing curcumin and β-CD showed greater antioxidant capacity and improved viscosity as well as stability, but presented a low rate of antimicrobial activity	[103]
β-CDs	Ceria NPs (CeNPs), natural antioxidant enzymes		Increase biocompatibility, water solubility and antioxidant capacity, anti-psoriatic effects.	[104]
β-CD	Babchi Oil—BO	Nanostructure gel	Reduce oxidative stress, anti-psoriatic effects	[105]
HPγCD	Curcumin	Hydrogel film	Wound healing	[106]

## Data Availability

Data sharing is not applicable.
